# 17,18-epoxyeicosatetraenoic acid ameliorates mRNA-LNP–induced local inflammation by inhibiting neutrophil infiltration

**DOI:** 10.1016/j.jlr.2025.100956

**Published:** 2025-12-05

**Authors:** Keigo Iemitsu, Ken Yoshii, Yuki Hirayama, Zilai Liu, Seiya Hifumi, Saki Kondo, Kei Ishida, Takahiro Nagatake, Jun Kunisawa

**Affiliations:** 1 Laboratory of Vaccine Materials and Laboratory of Gut Environmental System, Microbial Research Center for Health and Medicine, National Institutes of Biomedical Innovation, Health, and Nutrition (NIBN), Osaka, Japan; 2 Graduate School of Medicine, The University of Osaka, Osaka, Japan; 3 Graduate School of Pharmaceutical Sciences, The University of Osaka, Osaka, Japan; 4 Faculty of Health and Medical Sciences, Department of Sports and Health Sciences, Aichi Shukutoku University, Nagakute, Aichi, Japan; 5 Laboratory of Functional Anatomy, Department of Life Sciences, School of Agriculture, Meiji University, Kawasaki, Kanagawa, Japan; 6 Graduate School of Science, The University of Osaka, Osaka, Japan; 7 International Research and Development Center for Mucosal Vaccines, The Institute of Medical Science, The University of Tokyo, Tokyo, Japan; 8 Graduate School of Medicine, Kobe University, Kobe, Japan; 9 Research Organization for Nano and Life Innovation, Waseda University, Tokyo, Japan; 10 Graduate School of Dentistry, The University of Osaka, Osaka, Japan

**Keywords:** lipid nanoparticle, mRNA-LNP vaccine, side effect, 17,18-epoxyeicosatetraenoic acid, G protein–coupled receptor 40

## Abstract

Lipid nanoparticles (LNPs) are a powerful technology for delivering nucleic acids into cells and have greatly contributed to the development of severe acute respiratory syndrome coronavirus 2 mRNA vaccines and nucleic acid–based therapeutics. However, side effects such as pain and swelling at the injection site have been reported after vaccination with severe acute respiratory syndrome coronavirus 2 mRNA, and these are thought to be partly due to LNP-induced inflammation. In this study, we focused on an anti-inflammatory metabolite derived from omega-3 fatty acids and investigated whether it could suppress LNP-induced inflammatory side effects associated with mRNA-LNP vaccination. Intramuscular injection of empty LNPs lacking mRNA elicited inflammatory responses in mice comparable to those induced by mRNA-LNPs. Moreover, neutrophil depletion with an antibody demonstrated that neutrophils are key effector cells in LNP-induced inflammation. To suppress this response, we focused on 17,18-epoxyeicosatetraenoic acid (17,18-EpETE), an omega-3 fatty acid metabolite known to target neutrophils. Intramuscular co-injection of empty LNPs and 17,18-EpETE significantly reduced local swelling and infiltration of immune cells, including neutrophils, at the injection site. Further analysis revealed that this anti-inflammatory effect of 17,18-EpETE was mediated via G protein–coupled receptor 40. Importantly, 17,18-EpETE did not impair antibody production elicited by mRNA-LNP vaccination. These findings suggest that 17,18-EpETE has potential as a supplementary agent to mitigate inflammatory side effects without compromising the immunogenic efficacy of mRNA-LNP vaccines.

## Graphical Abstract

**Figure:**
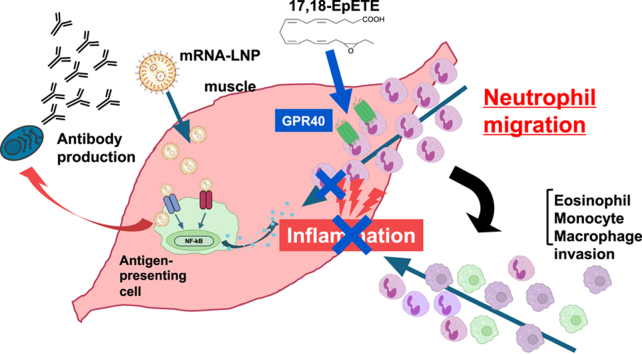

Lipid nanoparticles (LNPs) serve as an efficient intracellular delivery system for nucleic acid–based therapeutics, including mRNAs. This delivery platform has accelerated the development of nucleic acid–based treatments for a wide range of diseases, such as genetic disorders, cancer, and infectious diseases (1, 2, 3). Among these therapeutics, mRNA-LNP vaccines—where mRNAs encoding a pathogen-derived antigen are encapsulated in LNPs—have emerged as a promising new vaccine modality. The severe acute respiratory syndrome coronavirus 2 mRNA vaccine, developed to prevent coronavirus disease 2019, was the first mRNA-based drug approved by the US Food and Drug Administration and was subsequently authorized for use by regulatory agencies including the European Medicines Agency and Japan's Ministry of Health, Labour and Welfare.

mRNA-LNP vaccines consist of modified mRNA, where uridine residues are replaced with pseudouridine (4, 5, 6), and LNPs. The latter are formulated with functional lipids, including cationic lipids and polyethylene glycol, as well as structural lipids such as distearoylphosphatidylcholine and cholesterol (7). Unlike pathogens or their protein antigens, mRNAs are highly susceptible to degradation in vivo and are poorly taken up by cells when administered alone (8). Cationic lipids within LNPs bind to the mRNA and facilitate its encapsulation, thereby protecting it from enzymatic degradation. Upon endocytic uptake of mRNA-LNPs by cells, cationic lipids promote endosomal escape by disrupting the endosomal membrane, allowing the release of mRNA into the cytoplasm (9, 10). In addition to their role in mRNA delivery, cationic lipids can activate innate immune signaling via pattern recognition receptors, including toll-like receptors (TLRs) 2 and 4, thereby enhancing both humoral and cellular immune responses (11, 12, 13, 14, 15, 16).

While LNPs play an essential role in mRNA-LNP vaccines, as described above, they have also been implicated in the induction of inflammatory responses (11, 17, 18, 19). Such responses may contribute to injection site pain, headache, and fever in humans after vaccination (20, 21, 22). To mitigate these side effects, various strategies have been explored, including optimizing the lipid composition of LNPs, particularly that of cationic lipids (7, 16, 23), and incorporating anti-inflammatory agents such as the steroid drug dexamethasone into LNPs (24, 25). However, many of these studies lack comprehensive immunological evaluation, and steroid drugs may cause skin irritation and increase susceptibility to infections. Therefore, immunogenically effective and safe mRNA-LNP vaccines are needed; they have to elicit robust antigen-specific responses without triggering excessive inflammation.

Some anti-inflammatory fatty acid metabolites are generated through the enzymatic conversion of omega-3 (ω3) fatty acids such as α-linolenic acid—abundant in linseed and perilla oils—and marine-derived fatty acids including EPA and docosahexaenoic acid. These fatty acid metabolites are diverse structurally and functionally and exert anti-inflammatory and antiallergic effects through diverse mechanisms. We previously reported that a metabolite of EPA produced in the intestine, 17,18-epoxyeicosatetraenoic acid (17,18-EpETE), alleviates food allergies (26). In addition, it also inhibits allergic contact dermatitis by suppressing pseudopod formation in neutrophils via G protein–coupled receptor 40 (GPR40) on their surface (26, 27). We also identified another EPA-derived metabolite, produced by eosinophils in the nasal mucosa, 15-hydroxyeicosapentaenoic acid, attenuates allergic rhinitis by inhibiting mast cell degranulation (28). Moreover, we recently found that a unique metabolite produced by intestinal bacteria from α-linolenic acid, 10-oxo-*cis*-12-*cis*-15-octadecadienoic acid, activates peroxisome proliferator–activated receptor gamma and suppresses NF-κB activity in macrophages, improving glucose tolerance in adipose tissue and alleviating allergic contact dermatitis (29). In this study, we aimed to confirm the main mediators of LNP-induced inflammation and evaluate whether an ω3 fatty acid metabolite could mitigate side effects associated with mRNA-LNP vaccine administration.

## Materials and methods

### Mice

Wild-type C57BL/6J and BALB/c female mice (age, 7 weeks) were purchased from CLEA Japan (Tokyo, Japan) and kept in a specific pathogen-free animal facility at the National Institutes of Biomedical Innovation, Health, and Nutrition (Osaka, Japan) for 1 week before use in experiments. Mice were killed by cervical dislocation under anesthesia with isoflurane (Forane; AbbVie, North Chicago, I). All experiments were performed in accordance with the guidelines of the Animal Care and Use Committee and the Committee on the Ethics of Animal Experiments at National Institutes of Biomedical Innovation, Health, and Nutrition (approval nos. DSR04-37R7 and DSR02-29R2).

### LNP-induced inflammation

The hair from the leg skin of isoflurane-anesthetized mice was shaved with an electric clipper. The mice were injected into the gastrocnemius with 100 μl of the following suspensions (all in PBS; Nacalai Tesque, Kyoto, Japan): PBS only, empty LNPs (eLNPs, OZ Biosciences, Marseille, France) (0.125 μmol), or ovalbumin (OVA) mRNA-LNPs (OZ Biosciences) (5 μg). In some experiments, mice were also injected into the gastrocnemius with 100 μl of the following suspensions (all in PBS containing 1% [vol/vol] ethanol; Nacalai Tesque): PBS only, eLNP (0.125 μmol), 17,18-EpETE (Cayman Chemical, Ann Arbor, MI; 1 μg of 17,18-EpETE equivalent to 0.0031 μmol in 100 μl of vehicle, or a concentration of 31 μM) and eLNP (0.125 μmol), or 17,18-dihydroxy-eicosa-5,8,11,14-tetraenoic acid (17,18-diHETE) (Cayman Chemical; 1 μg of 17,18-diHETE equivalent to 0.0030 μmol in 100μl of vehicle, or a concentration of 30 μM) and eLNP (0.125 μmol). In some experiments, mice were first injected peritoneally with 100 μl of the following suspensions (all in PBS containing 1% [vol/vol] ethanol): PBS only, 17,18-EpETE (1 μg) and then were injected into the gastrocnemius with 100 μl of the following suspensions (all in PBS containing 1% [vol/vol] ethanol): PBS only, eLNPs (0.125 μmol), 17,18-EpETE (1 μg), and eLNP (0.125 μmol). At 4, 8, 16, 24 h after injection, the triceps surae and plantaris were collected by cutting the heel and Achilles tendon, and the soleus and plantaris were separated from the gastrocnemius to harvest the latter. To identify target receptors for 17,18-EpETE, mice were injected intraperitoneally with the GPR40 antagonist GW1100 (ChemScene, Monmouth Junction, NJ) (1 mg/kg), GPR120 antagonist AH7614 (ChemScene) (5 μg), or both 30 min before injection of a mixture of 17,18-EpETE and LNP. In experiments to deplete neutrophils, mice were injected intraperitoneally with the neutrophil-eliminating antibody Nimp-R14 (AdipoGen Life Sciences, San Diego, CA) (250 μg per mouse) 24 h before the injection of eLNPs. To assess the metabolism of 17,18-EpETE after intramuscular injection and its concentrations in the gastrocnemius muscle and in the serum, mice were injected into the gastrocnemius with 17,18-EpETE (1 μg).

### Cell isolation and flow cytometric analysis

The collected gastrocnemius muscles were cut into small pieces with scissors and incubated with 2.5 mg/ml collagenase (Wako, Tokyo, Japan) in RPMI 1640 medium (Sigma-Aldrich, St. Louis, MO) containing 2% (vol/vol) newborn calf serum (Serana, Brandenburg, Germany) with stirring (90 min, 37°C, 5% CO_2_). Cell suspensions were filtered through 100 μm cell strainers (Corning, New York, NY) and centrifuged (10 min, 4°C, 1400 rpm) to collect cells.

Cells within the gastrocnemius were stained with an anti-CD16/32 monoclonal antibody (mAb) (dilution ratio 1:100, TruStain fcX; BioLegend, San Diego, CA) to avoid nonspecific staining. Dead cells were detected with 7-aminoactinomycin D (1:100; 420404, BioLegend) and excluded from the analysis. The following fluorescently labeled mAbs (all from BioLegend) were used for flow cytometric analysis: FITC-anti-Ly6G (1:100, 127606, 1A8), phycoerythrin-anti-Ly6C (1:100, 128007, HK1.4), allophycocyanin-anti-CD45 (1:100, 103112, 30-F11), allophycocyanin-cyanine (Cy) 7-anti-CD11b (1:100; 101226, M1/70), phycoerythrin-Cy7-anti-F4/80 (1:100; 123114, BM8), and Brilliant Violet 421-anti-Siglec-F (1:100, 155509, E50-2440). Flow cytometric analysis was conducted on a CytoFLEX LX (Beckman Coulter, Brea, CA) or MACSQuant instrument (Miltenyi Biotec, Bergisch Gladbach, Germany). Data were analyzed in FlowJo 10.10.0 (Tree Star, Ashland, OR).

### Histological analysis

Histological analysis was performed as described previously (27) with a modification. Briefly, the gastrocnemius samples were frozen by directly immersing in isopentane supercooled with liquid nitrogen and stored at −80°C. Frozen gastrocnemius sections (8 μm) were cut on a cryostat (model CM3050 S, Leica Biosystems, Wetzlar, Germany) and used for hematoxylin and eosin staining and immunohistological analysis. The following antibodies were used: purified anti-Ly6G mAb (1:100, 127602, 1A8, BioLegend), purified anti-laminin (1:100, AB11575, Abcam, Cambridge), Alexa Fluor 488–anti-rat IgG (1:200, A-11006, Thermo Fisher Scientific, Waltham, MA), and Cy3-anti-rabbit IgG (Jackson ImmunoResearch Laboratories, West Grove, PA). Cell nuclei were stained with 4′,6-diamidino-2-phenylindole (1 mmol/L; AAT Bioquest, Sunnyvale, CA). Stained sections were examined under a fluorescence microscope (model BZ-9000; Keyence, Osaka, Japan).

### Lipid extraction and LC-MS/MS analysis

LC-MS/MS lipidomics analysis was performed as reported previously (30) with a modification. Briefly, prostaglandin E_2_ (PGE_2_), 17,18-EpETE, and 17,18-diHETE from the gastrocnemius or serum were separated on a MonoSpin C18-AX centrifugal column (GL Sciences, Tokyo, Japan) with deuterium-labeled internal standards. PGE_2_ was analyzed on a Shimadzu LCMS-8060NX system with a triple-quadrupole mass spectrometer (Shimadzu, Kyoto, Japan). Chromatographic separation used a Kinetex C8 column (2.1 × 150 mm, 2.6 μm; Phenomenex, Torrance, CA). Solvent A was 0.1% acetic acid, solvent B was acetonitrile. LC-MS data were analyzed in LabSolutions Insight (Shimadzu).

### Quantitative reverse-transcription PCR analysis

The analysis was performed as described previously (31) with a modification. Briefly, the gastrocnemius samples were homogenized in lysis buffer (LBA buffer with 2% 1-thioglycerol). The samples were centrifuged (3 min, room temperature [RT], 14,000 × *g*), and total RNA was isolated from the supernatant using a ReliaPrep RNA Tissue Miniprep System (Promega, Tokyo, Japan). Total RNA was incubated with DNase I (Invitrogen, Carlsbad, CA), and a Super Script VILO cDNA Synthesis kit (Invitrogen) was used to prepare cDNA. A CFX Opus Real-Time PCR System (Bio-Rad Laboratories, Hercules, CA) with PrimePCR Probe Assay was used for quantitative reverse-transcription PCR analysis (qRT-PCR). The unique assay IDs of PrimePCR Probes (Bio-Rad) for the CFX Opus Real-Time PCR System were as follows: *Tnfα* (qMmuCEP0028054), *Il6* (qMmuCEP0054186), *Il1b* (qMmuCEP0054181), *Cox2* (qMmuCEP0055740), *Icam1* (qMmuCEP0056911), *Vcam1* (qMmuCEP0055934), and *Actb* (qMmuCEP0039589).

### Immunization

The legs of isoflurane-anesthetized mice were shaved with an electric clipper, and the mice were injected into the gastrocnemius twice with a 2-week interval with 100 μl of the following suspensions (all in PBS containing 1% (vol/vol) ethanol): PBS only, OVA mRNA-LNPs (5 μg), or 17,18-EpETE (1 μg) and OVA mRNA-LNPs (5 μg). Blood was collected 2 weeks after the second immunization and centrifuged (10 min, 4°C, 3000 × *g*) to collect serum. Body temperature was obtained by measuring the rectal temperature. Body temperature and weight were monitored at 0, 1, 4, 8, 12, 24, and 48 h after the second immunization.

### Detection of OVA-specific antibodies by ELISA

Flat-bottom 96-well plates (Thermo Fisher Scientific) were coated with 1 mg/ml of OVA (Sigma-Aldrich) in PBS (100 μl/well) by incubating at 4°C overnight. To avoid nonspecific binding, the plates were then blocked with 1% BSA (Nacalai Tesque) in PBS (170 μl/well, RT, 2 h) and washed three times with wash buffer (PBS containing 0.05% Tween-20 (Nacalai Tesque)). Samples serially diluted with PBS containing 1% BSA and 0.05% Tween-20 were added to the plates (100 μl/well) and incubated at RT for 2 h. The plates were washed three times with wash buffer and incubated with horseradish peroxidase–conjugated goat antibodies against mouse IgG, IgG1, IgG2b, IgG2c, or IgG3 (SouthernBiotech, Birmingham, AL) diluted 1:4000 in PBS containing 0.05% Tween-20 and 1% BSA (100 μl/well, RT, 1 h). The plates were washed three times with wash buffer and incubated with tetramethylbenzidine peroxidase substrate (SeraCare Life Sciences, Milford, MA) (100 μl/well, RT, 2 min), and 0.5 M HCl (Nacalai Tesque) was added (50 μl/well) to stop the chromogenic reaction. Absorbance at 450 nm (A_450_) was measured in an iMark Microplate Absorbance Reader (Bio-Rad Laboratories).

### Statistics

In comparisons of multiple groups, statistical significance was evaluated by one-way ANOVA in Prism 10.3.0 software (GraphPad Software, La Jolla, CA). *P* values < 0.05 were considered significant.

## Results

### LNPs in mRNA-LNP vaccines induce local inflammatory responses at the injection site

**Fig. 1:**
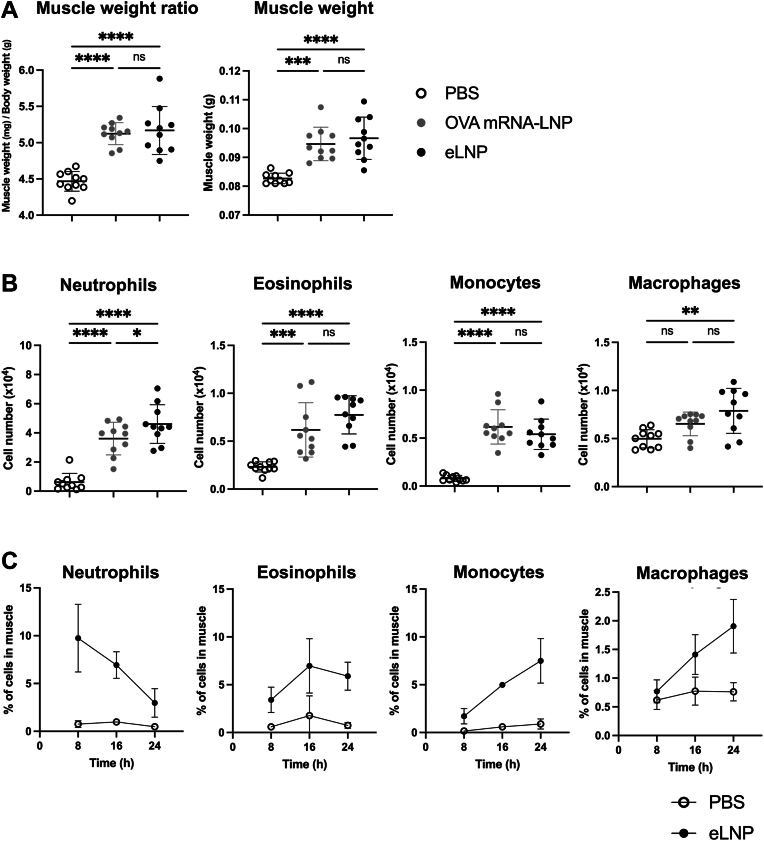
Gastrocnemius swelling and immune cell infiltration induced by LNPs. (A), (B) OVA mRNA-LNPs, eLNPs, or PBS (control) were injected into the gastrocnemius muscle of C57BL/6J mice. A: Weight of the gastrocnemius and relative weight of the gastrocnemius collected 4 h after injection. B: Flow cytometric quantification of immune cells isolated from the gastrocnemius. Gating strategy: neutrophils, CD45^+^ CD11b^+^ Ly6G^+^; eosinophils, CD45^+^ CD11b^+^ Ly6G^-^ Siglec-F^+^; monocytes, CD45^+^ CD11b^+^ Ly6G^-^ Siglec-F^-^ Ly6C^+^; macrophages, CD45^+^ CD11b^+^ Ly6G^-^ Siglec-F^-^ Ly6C^-^ F4/80^+^. Immune cell numbers were calculated from total cell numbers and flow cytometry data. In (A) and (B), data are from two independent experiments; each point represents an individual mouse. Data are mean ± SD. Statistical significance was determined by one-way ANOVA; ∗*P* < 0.05, ∗∗*P* < 0.01, ∗∗∗*P* < 0.001, and ∗∗∗∗*P* < 0.0001, ns, not significant. C: eLNP or PBS (control) was injected into the gastrocnemius of BALB/c mice (n = 4/group), and the gastrocnemius was collected at 8, 16, and 24 h. Flow cytometric quantification of immune cells isolated from the gastrocnemius. Each cells were gated as in Fig. 1B. Data are mean ± SD. LNP, lipid nanoparticle; eLNP, empty LNP; OVA, ovalbumin.

To evaluate the contribution of LNPs to this inflammatory response, we injected either LNPs containing OVA-encoding mRNA or eLNPs lacking mRNA into the mouse gastrocnemius muscle. At 4 h after injection, the gastrocnemius was collected and weighed to assess swelling as a readout for local inflammation. Gastrocnemius weight was significantly higher both in the OVA mRNA-LNP and eLNP groups than in the control group, with no appreciable difference between the former two groups (Fig. 1A). In flow cytometric analysis of immune cells in the muscle tissue (Supplemental Fig. S2), infiltration of neutrophils, eosinophils, and monocytes was higher in both the OVA mRNA-LNP and eLNP groups than in the control, along with a trend toward higher macrophage numbers (Fig. 1B). Gastrocnemius swelling persisted at least up to 24 h after eLNP injection (Supplemental Fig. S1A), with an early influx of neutrophils that gradually declined, while the numbers of eosinophils, monocytes, and macrophages increased progressively over time (Fig. 1C).

Throughout the 2 days after the second immunization, body weight and body temperature did not differ significantly between the OVA mRNA-LNP and control groups (Supplemental Fig. S1B). These findings indicate that, under the current experimental conditions, LNPs induced localized inflammatory responses at the injection site, but no detectable systemic effects.

### LNP-induced inflammatory responses are mediated by neutrophils

**Fig. 2:**
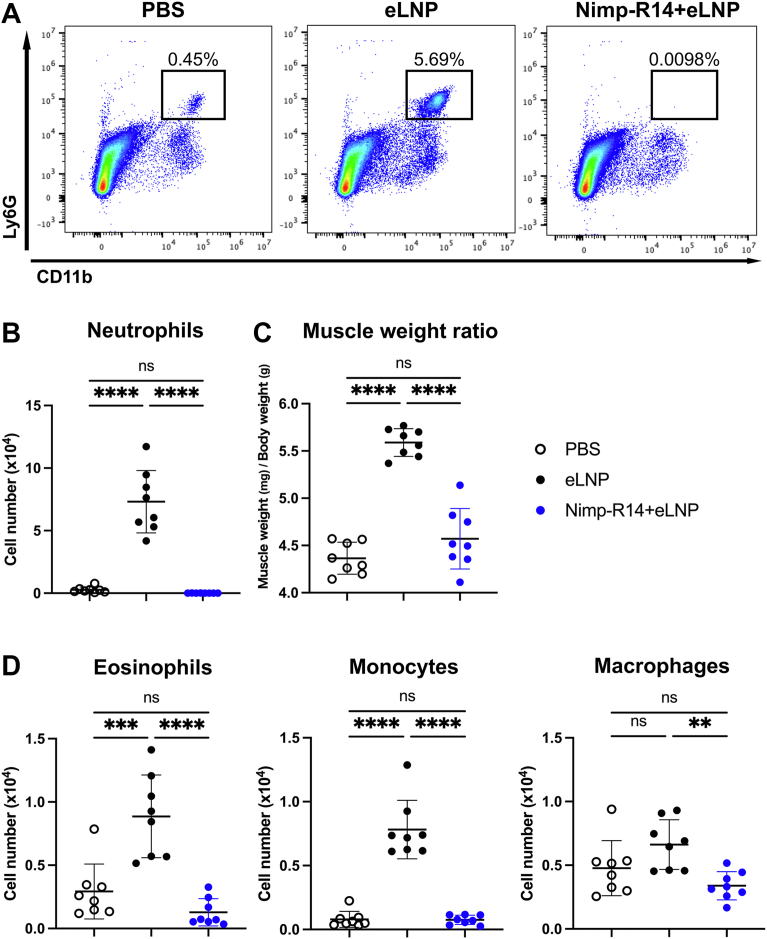
Neutrophils are essential mediators of LNP-induced inflammation. Neutrophil-depleting antibody Nimp-R14 or PBS (control) was intraperitoneally injected into C57BL/6J mice; 24 h later, eLNP or PBS was injected into the gastrocnemius. A: Representative flow cytometry plots showing CD11b^+^ Ly6G^+^ neutrophils from one of the two independent experiments. Percentages indicate the frequency of neutrophils among CD11b^+^ cells. B: Flow cytometric quantification of neutrophils. Immune cells were isolated from the gastrocnemius; neutrophils were gated as in Fig. 1B. C: Relative weight of the gastrocnemius collected 4 h after eLNP administration. D: Flow cytometric quantification of eosinophils, monocytes, and macrophages. Each cells were gated as in Fig. 1B. In (A), (C), and (D), data are from two independent experiments; each point represents an individual mouse. Data are mean ± SD. Statistical significance was determined by one-way ANOVA; ∗∗*P* < 0.01, ∗∗∗*P* < 0.001, and ∗∗∗∗*P* < 0.0001, ns, not significant. LNP, lipid nanoparticle; eLNP, empty LNP.

Intramuscular administration of both mRNA-LNPs and eLNPs led to an increase in inflammatory cells in the gastrocnemius muscle, with neutrophils being the predominant early responders (Fig. 1B, C). To evaluate the role of neutrophils in LNP-induced inflammation, we injected eLNPs into mice that had been intraperitoneally injected with the neutrophil-depleting antibody Nimp-R14. In flow cytometric analysis, Nimp-R14 effectively eliminated neutrophils from the gastrocnemius (Fig. 2A, B). At 4 h after eLNP injection, gastrocnemius swelling was significantly higher in the eLNP group than in the control group, whereas this response was absent in the neutrophil-depleted group (Fig. 2C; Supplemental Fig. S3). The numbers of eosinophils and monocytes were significantly higher and those of macrophages tended to be higher in the eLNP group than in the control, but no differences from the control were observed in the neutrophil-depleted group (Fig. 2D). These findings indicate that neutrophils are essential mediators of the inflammatory response induced by LNPs.

### 17,18-EpETE suppresses LNP-induced inflammatory responses

**Fig. 3:**
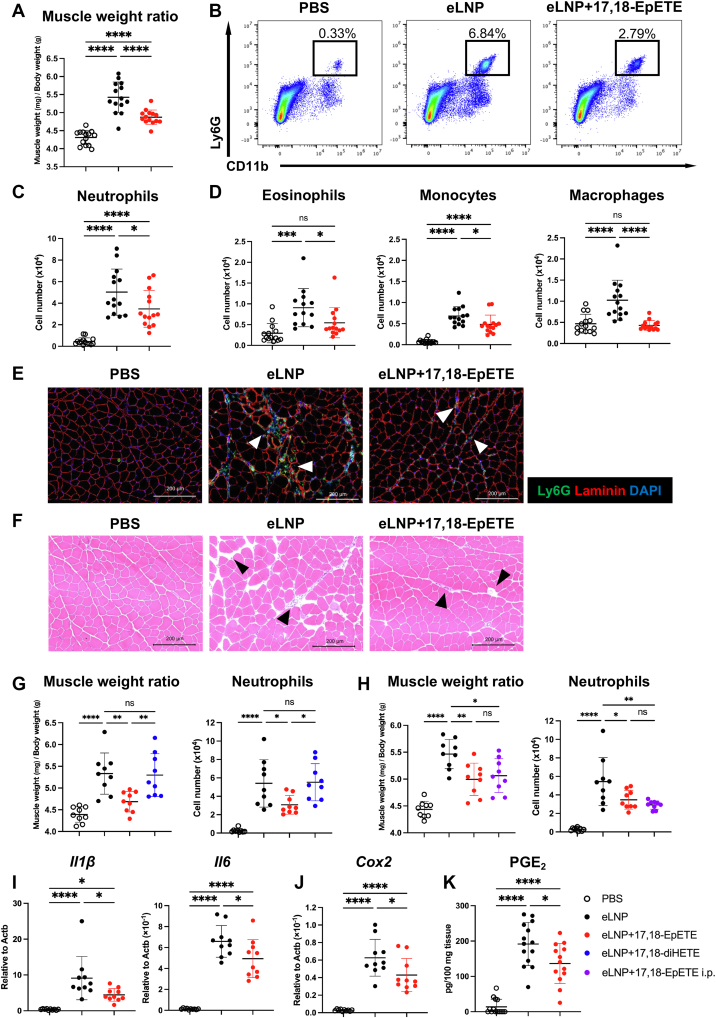
17,18-EpETE suppresses LNP-induced inflammatory responses. eLNPs or eLNPs with 17,18-EpETE or PBS (all in PBS containing 1% ethanol) were injected into the gastrocnemius of C57BL/6J mice. A: Relative weight of the gastrocnemius collected 4 h after injection. B: Representative flow cytometry plots showing CD11b^+^ Ly6G^+^ neutrophils from one of the four independent experiments. C, D: Flow cytometric quantification of immune cells. Each cells were gated as in Fig. 1B. E and F: Immunofluorescence (E) and histological (F) analyses of frozen gastrocnemius sections. Scale bars represent 200 μm. G and H: Relative weight of the gastrocnemius collected 4 h after injection and flow cytometric quantification of neutrophils. In (G), an additional group was included in which eLNPs with 17,18-diHETE were injected into the gastrocnemius; in (H), PBS alone or 17,18-EpETE was administered intraperitoneally. Immune cells were isolated from the gastrocnemius; neutrophils were gated as in Fig. 1B. I and J: Gene expression of *Il1β*, *Il6*, and *Cox2* in the gastrocnemius measured by qRT-PCR. All data were normalized to *Actb*. K: PGE_2_ concentrations in gastrocnemius tissue measured by LC-MS/MS. Data are from two (G–J), three (K), or four (A, C, and D) independent experiments; each point represents an individual mouse. Data are mean ± SD. Statistical significance was determined by one-way ANOVA; ∗*P* < 0.05, ∗∗*P* < 0.01, ∗∗∗*P* < 0.001, and ∗∗∗∗*P* < 0.0001. LNP, lipid nanoparticle; eLNP, empty LNP; 17,18-diHETE, 17,18-dihydroxy-eicosa-5,8,11,14-tetraenoic acid; 17,18-EpETE, 17,18-epoxyeicosatetraenoic acid; PGE_2_, prostaglandin E_2_; qRT-PCR, quantitative reverse-transcription PCR analysis.

Given that these inflammatory responses are driven by neutrophil infiltration into the injection site, we investigated the anti-inflammatory potential of 17,18-EpETE, an ω3 fatty acid–derived metabolite known to inhibit neutrophil pseudopod formation and migration (27). We co-injected 17,18-EpETE and eLNPs into the gastrocnemius. At 4 h after injection, muscle swelling was significantly lower in the 17,18-EpETE–treated group than in the eLNP-only group (Fig. 3A; Supplemental Fig. S4A). In flow cytometric analysis, the numbers of neutrophils (Fig. 3B, C), eosinophils, monocytes, and macrophages (Fig. 3D) in the gastrocnemius were lower in the 17,18-EpETE–treated group than in the eLNP-only group. Immunohistochemical staining of frozen sections confirmed the reduction of neutrophil infiltration in the presence of 17,18-EpETE (Fig. 3E). In histological analysis, the tissue gap expansion typically observed—indicative of inflammation in skeletal muscles (32)—was absent when 17,18-EpETE was co-injected (Fig. 3F). As stated in previous studies, epoxide hydrolase hydrolyzes 17,18-EpETE to form 17,18-diHETE (27). Indeed, 17,18-EpETE was rapidly metabolized to 17,18-diHETE in the gastrocnemius muscle after intramuscular injection (Supplemental Fig. S4B). However, in agreement with our previous findings of the absence of the effect of 17,18-diHETE on intestinal allergy and contact hypersensitivity (26, 27), 17,18-diHETE did not inhibit LNP-induced inflammation (Fig. 3G; Supplemental Fig. S4C, D). Furthermore, we confirmed the efficacy of 17,18-EpETE administered intraperitoneally (Fig. 3H; Supplemental Fig. S4E, F). To further assess the anti-inflammatory activity of 17,18-EpETE, we performed qRT-PCR analysis of the gastrocnemius muscle. This analysis demonstrated a significant downregulation of the expression levels of *Il1β, Il6,* and *Cox2* in the 17,18-EpETE–treated group (Fig. 3I, J). We also examined the expression levels of *Icam1* and *Vcam1*, which are involved not only in promoting immune cell recruitment but also in tissue repair in skeletal muscle (33, 34), and found no differences between the 17,18-EpETE–treated group and the eLNP-only group (Fig. S4G). Similarly, no difference was observed in the expression level of *Tnfα*, a major cytokine that induces *Icam1* and *Vcam1* expression (Supplemental Fig. S4H). In LC-MS/MS analysis, the local concentration of PGE_2_, a key inflammatory mediator responsible for swelling, pain, and fever (35), was significantly lower in the 17,18-EpETE–treated group than in the eLNP-only group (Fig. 3K). These findings indicate that 17,18-EpETE effectively suppresses LNP-induced local inflammation.

### 17,18-EpETE suppresses LNP-induced inflammatory responses via GPR40

17,18-EpETE is a potent agonist of GPR40, capable of inhibiting allergic contact dermatitis by suppressing neutrophil infiltration through GPR40 signaling (27). It also acts as an agonist of GPR120 (27, 36) and inhibits liver fibrosis in mice via macrophage-expressed GPR120 (36).

**Fig. 4:**
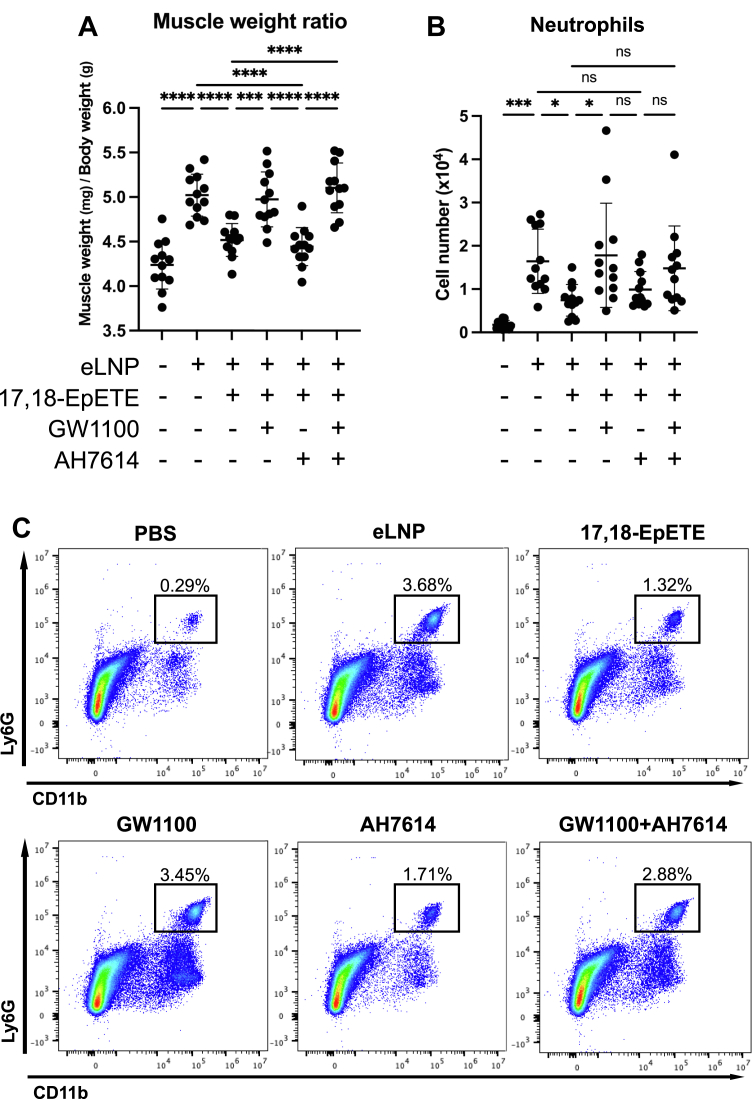
17,18-EpETE suppresses LNP-induced inflammation via GPR40 GPR40. antagonist GW1100, GPR120 antagonist AH7614, or PBS (all in PBS containing 1% DMSO) was intraperitoneally injected into C57BL/6J mice; 30 min later, eLNP, eLNP + 17,18-EpETE, or mock was injected into the gastrocnemius. A: Relative weight of the gastrocnemius collected 4 h after the intramuscular injection. B: Flow cytometric quantification of neutrophils (CD45^+^ CD11b^+^ Ly6G^+^). C: Representative flow cytometry plots showing CD11b^+^ Ly6G^+^ neutrophils from one of the four independent experiments. Data are from four independent experiments; each point represents an individual mouse. Data are mean ± SD. Statistical significance was determined by one-way ANOVA; ∗*P* < 0.05, ∗∗∗*P* < 0.001, and ∗∗∗∗*P* < 0.0001, ns, not significant. LNP, lipid nanoparticle; eLNP, empty LNP; 17,18-EpETE, 17,18-epoxyeicosatetraenoic acid; GPR, G protein–coupled receptor.

To determine which receptor mediates the anti-inflammatory effects of 17,18-EpETE in the context of LNP-induced inflammation, we pretreated mice with either the selective GPR40 antagonist GW1100 or with the GPR120 antagonist AH7614, followed by co-injection of eLNPs and 17,18-EpETE into the gastrocnemius. At 4 h after injection, the anti-inflammatory effect of 17,18-EpETE—as evidenced by reduced muscle swelling—was abolished by GW1100 but not by AH7614 (Fig. 4A; Supplemental Fig. S5A). In flow cytometric analysis, the suppressive effect of 17,18-EpETE on neutrophil accumulation was abolished by GW1100 (Fig. 4B, C), and similar trends were observed for eosinophils, monocytes, and macrophages (Supplemental Fig. S5B), whereas AH7614 had no effect. These results demonstrate that the anti-inflammatory effects of 17,18-EpETE on LNP-induced immune responses are mediated primarily through activation of GPR40.

### 17,18-EpETE does not impair antigen-specific antibody responses induced by mRNA-LNP vaccination

**Fig. 5:**
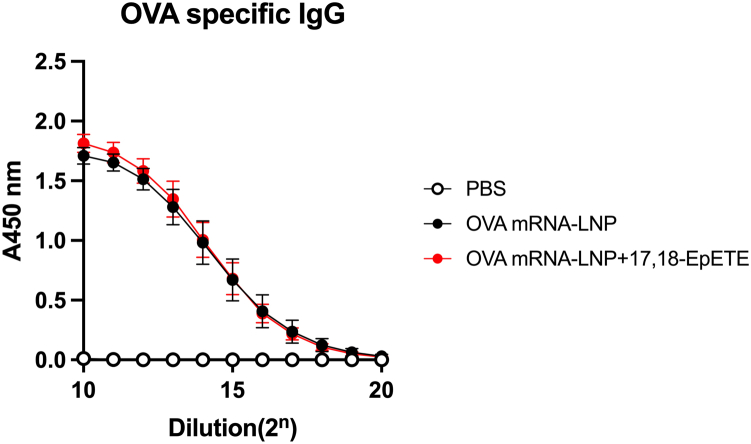
17,18-EpETE does not impair vaccine efficacy OVA mRNA-LNPs with or without 17,18-EpETE or PBS (control) were injected into the gastrocnemius of C57BL/6J mice. Two weeks after the second dose, serum OVA-specific IgG levels were measured by ELISA. Data are pooled from two independent experiments and are mean ± SD. LNP, lipid nanoparticle; 17,18-EpETE, 17,18-epoxyeicosatetraenoic acid; OVA, ovalbumin.

Because LNPs function as adjuvants and enhance immunogenicity (11, 12, 18), we investigated whether 17,18-EpETE would affect vaccine efficacy by measuring antigen-specific antibody production. We injected OVA mRNA-LNPs with or without 17,18-EpETE into the gastrocnemius and assessed serum levels of OVA-specific antibodies 2 weeks after the final injection. The production of OVA-specific IgG antibodies was similar in both groups (Fig. 5). Similar results were observed for all measured IgG subclasses, namely IgG1, IgG2b, IgG2c, and IgG3 (Supplemental Fig. S6). These findings indicate that 17,18-EpETE suppressed LNP-associated inflammatory responses without compromising the antigen-specific immune response elicited by mRNA-LNP vaccines.

## Discussion

In this study, we demonstrated that 17,18-EpETE suppresses LNP-induced inflammatory responses in muscle—an underlying cause of vaccine-related side effects—through GPR40 signaling, without compromising the immunogenicity of an mRNA-LNP vaccine. Given that not only mRNA-LNP vaccines but also LNP-based nucleic acid therapeutics will likely be administered repeatedly over extended periods, ensuring their safety by minimizing inflammatory responses is critically important. We consider the approach presented in this study to be broadly applicable and effective. As 17,18-EpETE can be readily combined with existing LNP formulations, this strategy offers a practical and adaptable means of mitigating side effects of current and future LNP-based nucleic acid medicines.

We observed a significantly higher number of neutrophils in the gastrocnemius muscle in the eLNP group than in the OVA mRNA-LNP group and found similar trends for eosinophils and macrophages (Fig. 1B). This difference is likely attributable to variations in surface composition between the two nanoparticle formulations. Cationic lipids present in LNPs are recognized by TLR2 and TLR4 (13, 14, 15). LNPs containing cationic lipids but not those lacking them promote immune cell infiltration at the injection site (17). Encapsulation of mRNA within LNPs results in a less positively charged surface than that of eLNPs (37). Thus, it is plausible that, in mRNA-LNPs, a portion of the cationic lipids is incorporated into the particle interior along with the mRNA, leading to their lower density on the outer surface relative to that in eLNPs. This reduced surface availability of cationic lipids may explain the attenuated inflammatory response observed in the mRNA-LNP group.

Time-course analysis following intramuscular injection of eLNPs revealed that neutrophils infiltrated the tissue first, followed sequentially by an increase in eosinophils, and then an increase in monocytes and macrophages (Fig. 1C). In acute inflammation, neutrophils are the first responders that rapidly migrate into tissues in response to proinflammatory cytokines and chemokines. Once recruited, neutrophils amplify inflammation through phagocytosis and undergo apoptosis, after which the resolution phase begins as macrophages clear apoptotic neutrophils and cellular debris (38). Eosinophils appear early during the resolution phase and contribute to the production of anti-inflammatory lipid mediators by 12- and 15-lipoxygenases; these mediators act on neighboring cells to facilitate the rapid resolution of inflammation (39). Eosinophils, monocytes, and macrophages observed in this study may play a similar role in modulating and resolving the inflammatory response induced by LNPs.

To assess systemic inflammatory responses to mRNA-LNP vaccination, we monitored body temperature and body weight in mice after injection with OVA mRNA-LNPs and found that neither increased significantly in comparison with those in the control group (Supplemental Fig. S1B). Serum PGE_2_, a key mediator of fever and inflammation, was undetectable by LC-MS/MS analysis (unpublished data). In contrast, previous studies have reported marked body weight loss and elevated body temperature after administration of certain LNP formulations (17, 40). Since immune responses can vary depending on the lipid composition of LNPs (7, 16, 40), these results suggest that the OVA mRNA-LNPs used in this study elicit a local inflammatory response without inducing significant systemic inflammation. In addition, a dose-dependent decrease in body weight induced by mRNA-LNP administration has been reported in mice (17). Therefore, increasing the dose of OVA mRNA-LNPs would likely trigger systemic inflammatory responses, including weight loss and fever, potentially by elevating serum PGE_2_ levels.

After administration into the gastrocnemius muscle, 17,18-EpETE was rapidly metabolized to 17,18-diHETE (Supplemental Fig. S4B), but administration of 17,18-diHETE did not suppress LNP-derived inflammation (Fig. 3G; Supplemental Fig. S4C, D), suggesting that the observed anti-inflammatory effects were mediated by 17,18-EpETE itself, and indicating that it acts during the early phase of inflammation following intramuscular injection. Intraperitoneal administration of 17,18-EpETE also suppressed LNP-induced inflammation (Fig. 3H; Supplemental Fig. S4E, F). This finding suggests that 17,18-EpETE acts both locally and systemically. LC-MS/MS analysis revealed that 17,18-EpETE suppressed the production of PGE_2_ (Fig. 3K). PGE_2_ production is induced through activation of *Cox2* expression by inflammatory cytokines such as IL-1β and IL-6, and several studies have mentioned that PGE_2_ is involved in the adverse effects of mRNA–LNP vaccines. (41, 42). Therefore, we suggest that 17,18-EpETE suppresses *Cox2* expression by inhibiting *Il1β* and *Il6* expression, and that the downregulation of *Cox2* consequently reduces PGE_2_ production (Fig. 3I–K). Previous studies have reported that 17,18-EpETE suppresses the expression of *Icam1* and *Vcam1* (43), but qRT-PCR analysis revealed that their expression levels in the muscle were comparable between the 17,18-EpETE-treated group and the eLNP-only group (Supplemental Fig. S4G). Similarly, the expression level of *Tnfα* was not altered (Supplemental Fig. S4H). This may be due to the differences in the dose, administration route, or target tissue between the previous study (43) and the present experiments, which might have been insufficient to affect *Icam1* and *Vcam1* expression.

We found that 17,18-EpETE suppresses LNP-induced inflammation via GPR40 (Fig. 4; Supplemental Fig. S5). Some studies have demonstrated that 17,18-EpETE binds to GPR40 expressed on neutrophils and suppresses pseudopod formation by inhibiting Rac activation (27), whereas other reports have described contrasting effects. For example, the GPR40 agonist GW9508 reportedly enhances neutrophil chemotaxis and phagocytosis (44), and linoleic and oleic acids (both ω9 fatty acids) activate neutrophils via GPR40 (45). These findings suggest that GPR40 may suppress excessive inflammation while also enhancing host defense mechanisms against infection. The functional outcome of GPR40 activation likely depends on the specific ligand involved, as GPR40 can couple to different G protein α subunits (Gαs, Gαq, or Gαi), thereby activating distinct signaling pathways (46). Activation of Gαs increases intracellular cAMP, whereas that of Gαi reduces cAMP levels. Gαq activation elevates intracellular Ca^2+^ concentration. cAMP activates protein kinase A (PKA), whereas Ca^2+^ activates protein kinase C. Notably, PKA inhibits P-Rex1, the primary guanine nucleotide exchange factor for Rac, whereas protein kinase C enhances its activity (47). Our hypothesis based on this mechanistic framework is that 17,18-EpETE induces GPR40-mediated activation of the Gαs–cAMP–PKA axis, which in turn suppresses P-Rex1 activity and Rac-dependent neutrophil activation. In addition, 17,18-EpETE used in this study (Cayman Chemical) is a racemic mixture composed of both 17(*S*),18(*R*)- and 17(*R*),18(*S*)-enantiomers. According to previous findings, the 17(*S*),18(*R*)-EpETE form predominantly exerts anti-inflammatory activity via GPR40-mediated inhibition of neutrophil migration, whereas the 17(*R*),18(*S*)-EpETE form has weaker activity under identical experimental conditions (48). Therefore, the anti-inflammatory effects observed in this study are most likely attributable, at least in part, to the 17(*S*),18(*R*)-enantiomer.

Taken together, our findings suggest that immunomodulation using ω3 fatty acid metabolites such as 17,18-EpETE represents a promising strategy for the development of next-generation mRNA-LNP vaccines that are both safe and effective.

## Data Availability

The data supporting the findings of this study are available from the corresponding author upon reasonable request.

## Supplemental Data

This article contains supplemental data.

## Conflict of Interest

The authors declare that they have no conflicts of interest with the contents of this article.

## Supplemental Data

Supplemental Figures

**Abbreviations**
ω3, omega-3
17,18-diHETE, 17,18-dihydroxy-eicosa-5,8,11,14-tetraenoic acid
17,18-EpETE, 17,18-epoxyeicosatetraenoic acid
eLNP, empty LNP
GPR40, G protein–coupled receptor 40
LNP, lipid nanoparticle
mAb, monoclonal antibody
OVA, ovalbumin
PGE_2_, prostaglandin E_2_
PKA, protein kinase A
qRT-PCR, quantitative reverse-transcription PCR analysis
RT, room temperature
TLR, toll-like receptor
