# Supplementary figures and images for: 17,18-epoxyeicosatetraenoic acid ameliorates mRNA-LNP–induced local inflammation by inhibiting neutrophil infiltration
Source: J Lipid Res. 2025 Dec 5;67(1):100956. doi: 10.1016/j.jlr.2025.100956 (PMC12809493; doi:10.1016/j.jlr.2025.100956)

**A**

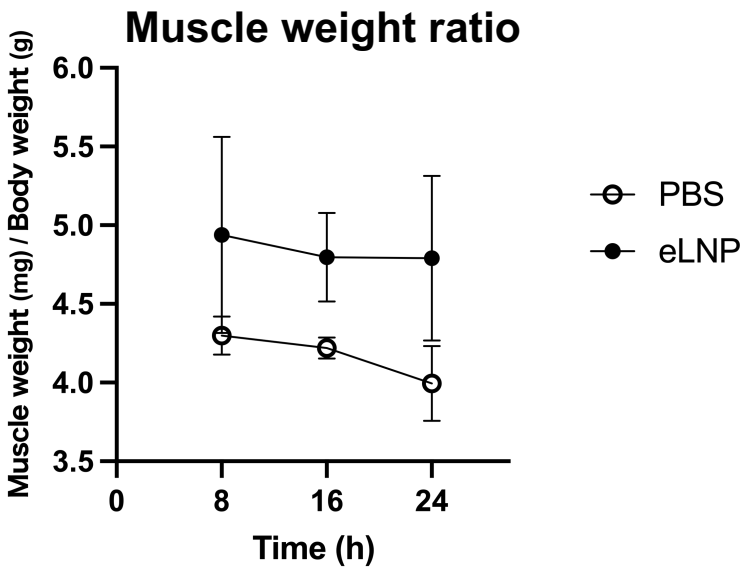

**B**

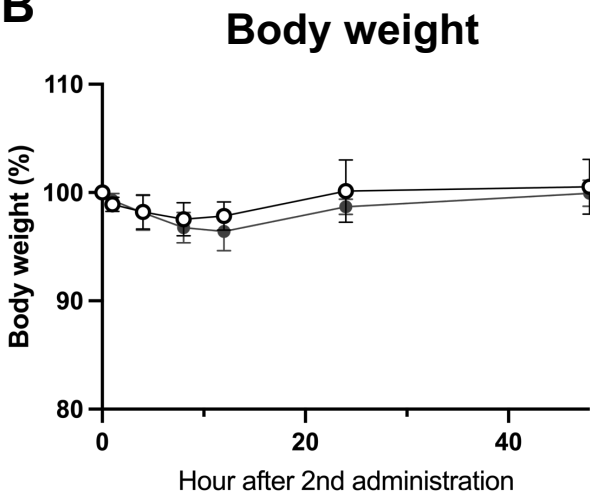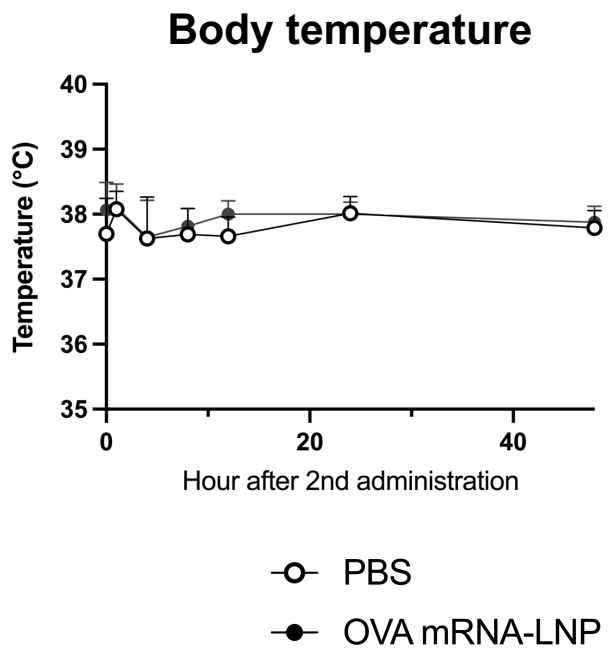

**Fig. S1**

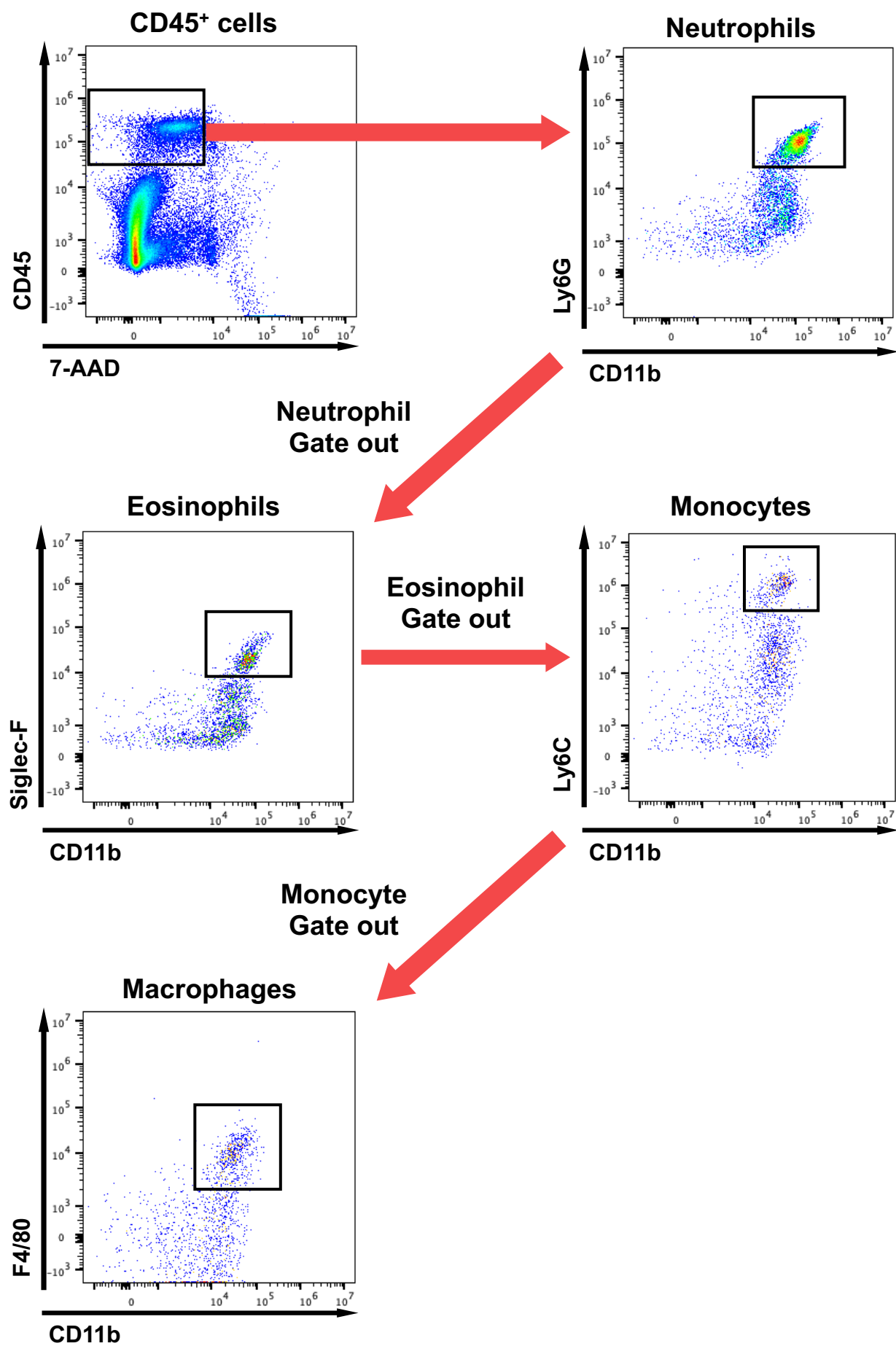

**Fig. S2**

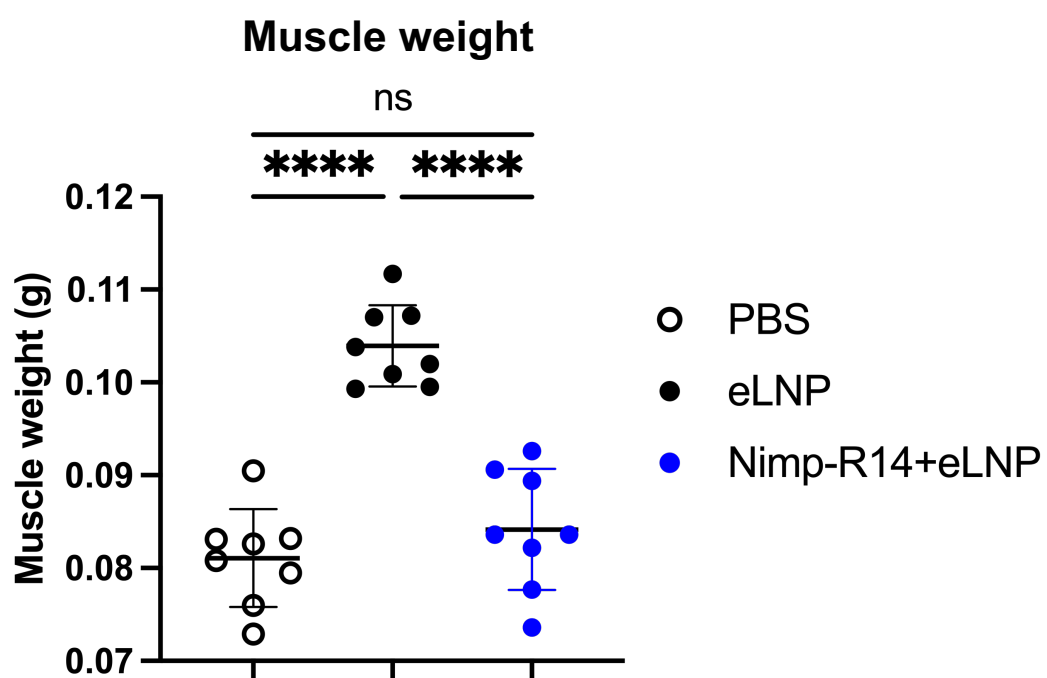

**Fig. S3**

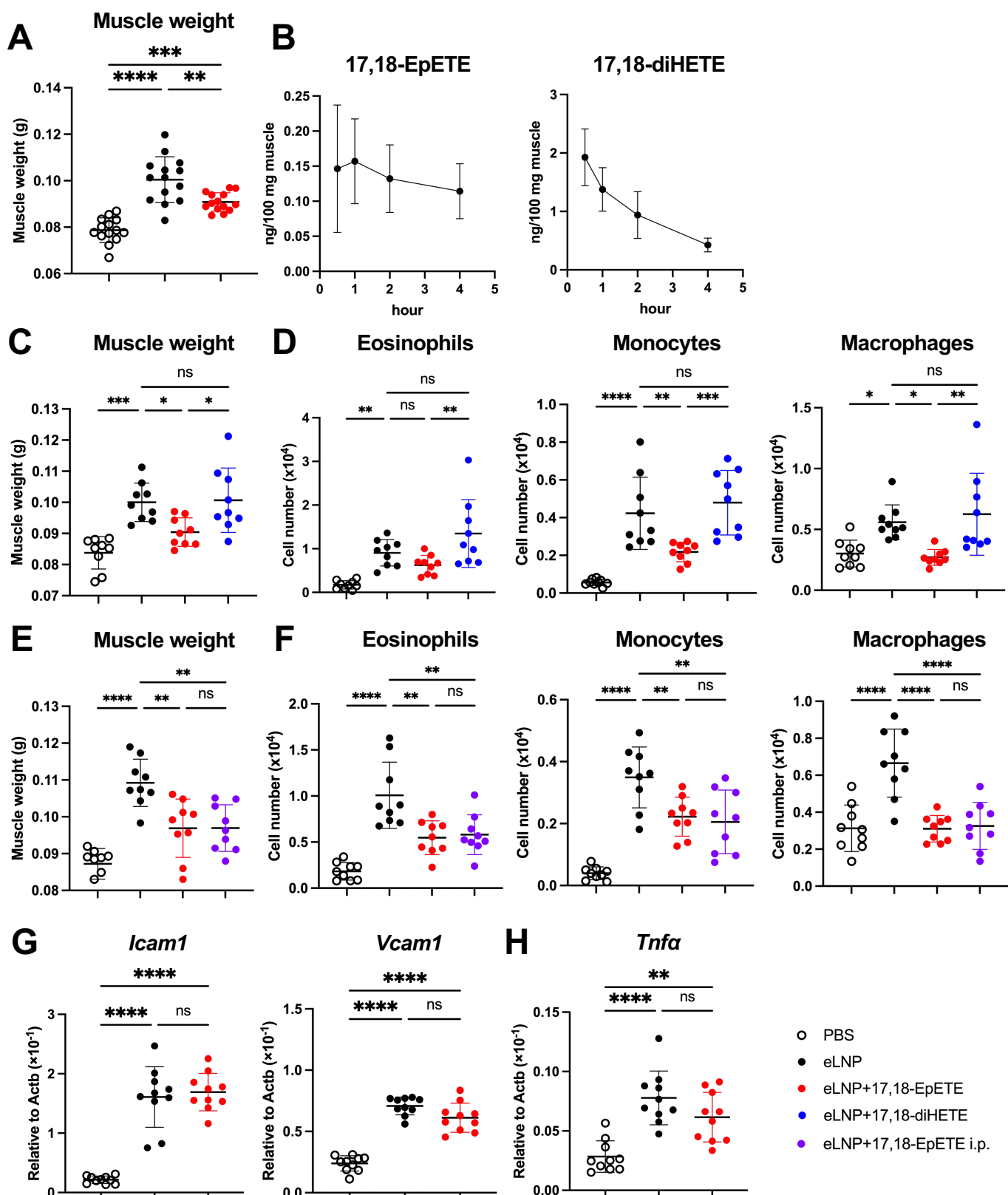

**Fig. S4**

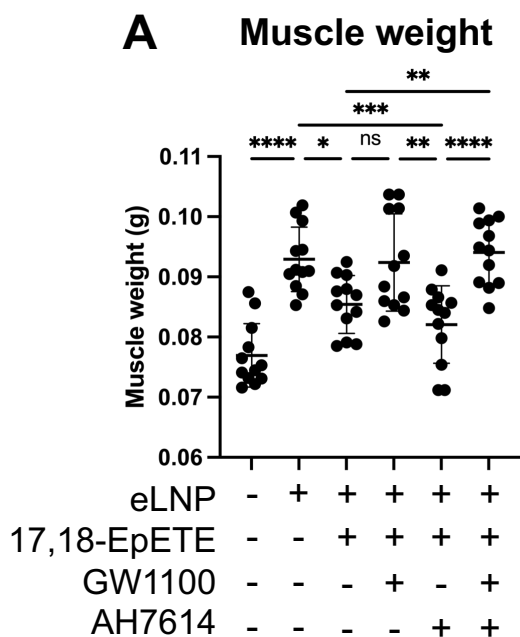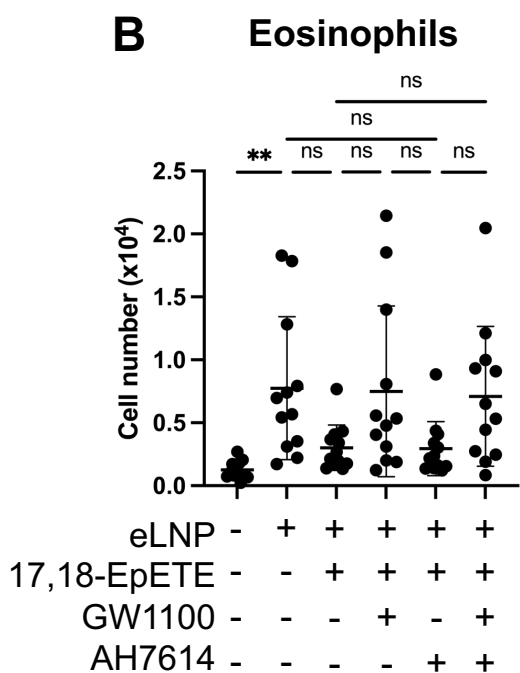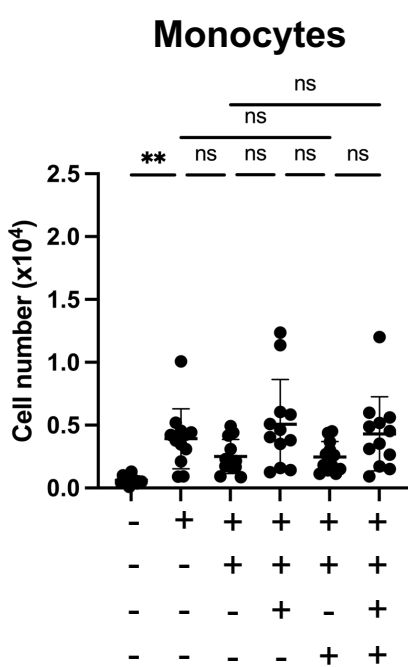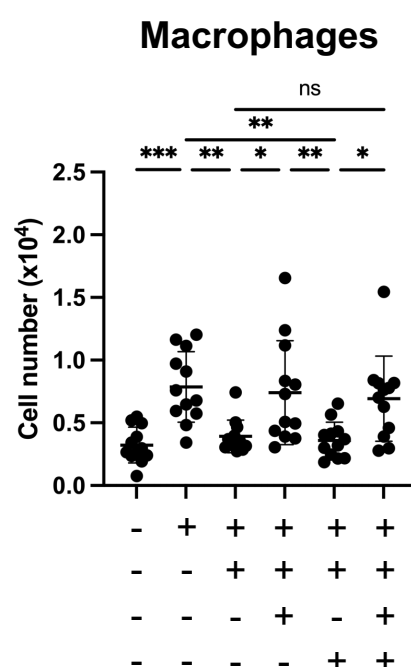

**Fig. S5**

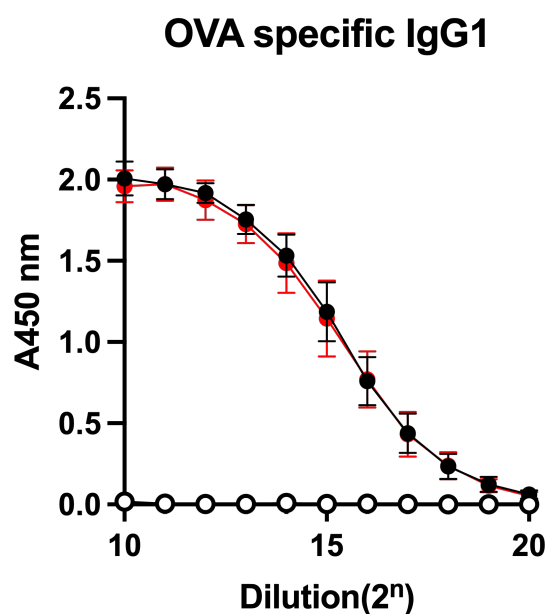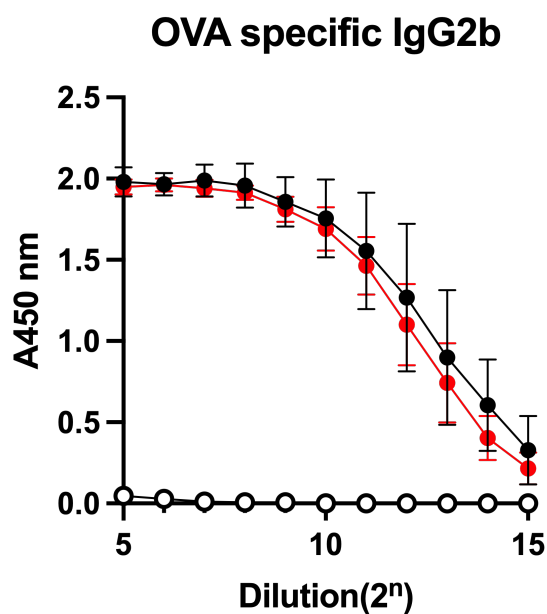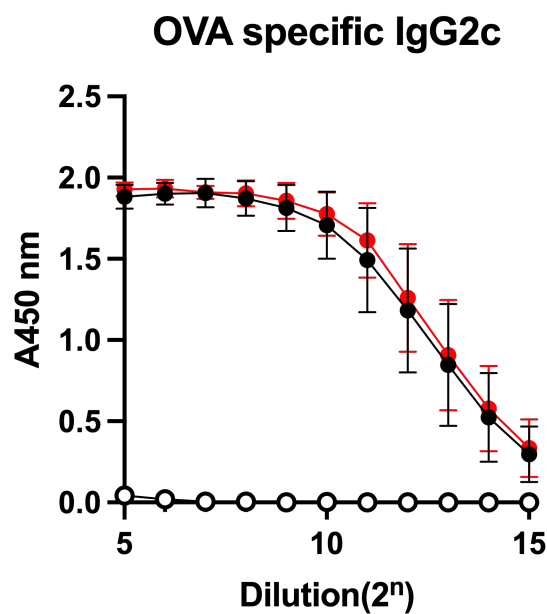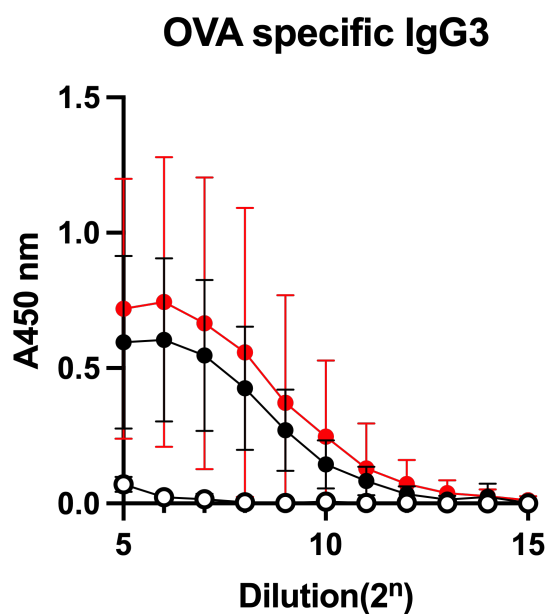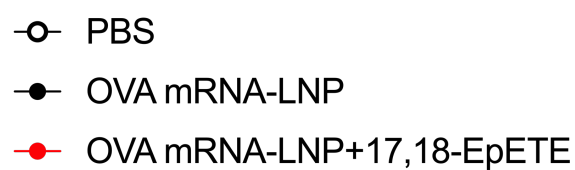

**Fig. S6**

Supplement: Supplemental Figures [file mmc1.pdf]
