# Supplementary material for: 17,18-epoxyeicosatetraenoic acid ameliorates mRNA-LNP–induced local inflammation by inhibiting neutrophil infiltration
Source: J Lipid Res. 2025 Dec 5;67(1):100956. doi: 10.1016/j.jlr.2025.100956 (PMC12809493; doi:10.1016/j.jlr.2025.100956)
Supplement: Supplemental Figures Legends [file mmc2.docx]

**Supplementary Figure 1** **Time course of LNP-induced inflammation**

(A) eLNP or PBS (control) was injected into the gastrocnemius of BALB/c mice (n = 4/group), and relative weight of the gastrocnemius was collected at 8, 16, and 24 h. at each time point. Data are mean ± SD. (B) OVA mRNA-LNPs in PBS or PBS (both containing 1% ethanol) was injected into the gastrocnemius of C57BL/6J mice twice with a 2-week interval. Body temperature and weight were monitored at 0, 1, 4, 8, 12, 24, and 48 h after the second immunization. Data are pooled from two independent experiments. Results are mean ± SD.

**Supplementary Figure 2 Gating strategy for gastrocnemius cells (related to Figure 1)**

Following injection of OVA mRNA-LNPs, eLNPs, or PBS (control) into C57BL/6J mice, the gastrocnemius was collected and processed for flow cytometry. Immune cells were gated as follows: neutrophils, CD45⁺ CD11b⁺ Ly6G⁺; eosinophils, CD45⁺ CD11b⁺ Ly6G⁻ Siglec-F⁺; monocytes, CD45⁺ CD11b⁺ Ly6G⁻ Siglec-F⁻ Ly6C⁺; macrophages, CD45⁺ CD11b⁺ Ly6G⁻ Siglec-F⁻ Ly6C⁻ F4/80⁺.

**Supplementary Figure 3** **Neutrophils promote infiltration of other immune cells in LNP-induced inflammation**

Neutrophil-depleting antibody Nimp-R14 or PBS (control) was intraperitoneally injected into C57BL/6J mice; 24 h later, eLNPs or PBS was injected into the gastrocnemius. Gastrocnemius weight at 4 h after eLNP injection. Data are from two independent experiments; each point represents an individual mouse. Data are mean ± SD. Statistical significance was determined by one-way ANOVA; *****p* < 0.0001, ns, not significant.

**Supplementary Figure 4** **17,18-EpETE suppresses LNP-induced inflammation and inhibits infiltration of non-neutrophil immune cells**

eLNPs or eLNPs with 17,18-EpETE or PBS (all in PBS containing 1% ethanol) were injected into the gastrocnemius of C57BL/6J mice. (A) Weight of the gastrocnemius harvested 4 h after injection. (B) 17,18-EpETE and 17,18-diHETE concentrations in the gastrocnemius measured by LC-MS/MS after injection of 17,18-EpETE. (C–F) Weight of the gastrocnemius harvested 4 h after injection and flow cytometric quantification of eosinophils, monocytes, and macrophages. Gating was as described above. In (C, D), an additional group was included in which eLNPs with 17,18-diHETE were injected into the gastrocnemius. In (E, F), PBS alone or 17,18-EpETE was administered intraperitoneally. (G, H) Gene expression of *Icam1*, *Vcam1*, and *Tnfα* in the gastrocnemius measured by qRT-PCR. All data were normalized to *Actb*. Data are from two (B-H) or four (A) independent experiments; each point represents an individual mouse. Data are mean ± SD. Statistical significance was determined by one-way ANOVA; **p* < 0.05, ***p* < 0.01, ****p* < 0.001, *****p* < 0.0001, ns, not significant.

**Supplementary Figure 5** **GPR40 inhibition abolishes 17,18-EpETE-mediated suppression of immune cell infiltration**

GPR40 antagonist GW1100, GPR120 antagonist AH7614, or PBS (all in PBS containing 1% DMSO) was injected intraperitoneally into C57BL/6J mice, followed 30 min later by injection of eLNPs, eLNPs + 17,18-EpETE, or PBS (all in PBS containing 1% ethanol) into the gastrocnemius. (A) Weight of the gastrocnemius harvested 4 h after the intramuscular injection. (B) Flow cytometric quantification of eosinophils, monocytes, and macrophages. Gating was as described above. Data are from four independent experiments; each point represents an individual mouse. Data are mean ± SD. Statistical significance was determined by one-way ANOVA; **p* < 0.05, ***p* < 0.01, ****p* < 0.001, *****p* < 0.0001, ns, not significant.

**Supplementary Figure 6** **17,18-EpETE does not interfere with mRNA-LNP vaccine–induced IgG subclass responses**

OVA mRNA-LNPs, OVA mRNA-LNPs + 17,18-EpETE, or PBS (all in PBS containing 1% ethanol) were injected into the gastrocnemius of C57BL/6J mice twice with a 2-week interval. Two weeks after the second injection, serum was collected and the levels of OVA-specific IgG subclasses were quantified by ELISA. Data are pooled from two independent experiments. Results are mean ± SD.
